# How can we strengthen partnership and coordination for health system emergency preparedness and response? Findings from a synthesis of experience across countries facing shocks

**DOI:** 10.1186/s12913-022-08859-6

**Published:** 2022-11-29

**Authors:** Kate Gooding, Maria Paola Bertone, Giulia Loffreda, Sophie Witter

**Affiliations:** 1 ReBUILD for Resilience and Oxford Policy Management, Clarendon House, Level 3, 52 Cornmarket Street, Oxford, OX1 3HJ UK; 2 ReBUILD for Resilience & Institute for Global Health and Development – Queen Margaret University, Edinburgh, EH21 6UU UK

**Keywords:** Resilience, Fragility, Shocks, Partnership, Coordination, Health systems

## Abstract

**Background:**

Discussions of health system resilience and emergency management often highlight the importance of coordination and partnership across government and with other stakeholders. However, both coordination and partnership have been identified as areas requiring further research. This paper identifies characteristics and enablers of effective coordination for emergency preparedness and response, drawing on experience from different countries with a range of shocks, including floods, drought, and COVID-19.

**Methods:**

The paper synthesises evidence from a set of reports related to research, evaluation and technical assistance projects, bringing together evidence from 11 countries in sub-Saharan Africa and South Asia. Methods for the original reports included primary data collection through interviews, focus groups and workshop discussions, analysis of secondary data, and document review. Reports were synthesised using a coding framework, and quality of evidence was considered for reliability of the findings.

**Results:**

The reports highlighted the role played by coordination and partnership in preparedness and response, and identified four key areas that characterise and enable effective coordination. First, coordination needs to be inclusive, bringing together different government sectors and levels, and stakeholders such as development agencies, universities, the private sector, local leaders and civil society, with equitable gender representation. Second, structural aspects of coordination bodies are important, including availability of coordination structures and regular meeting fora; clear roles, mandates and sufficient authority; the value of building on existing coordination mechanisms; and ongoing functioning of coordination bodies, before and after crises. Third, organisations responsible for coordination require sufficient capacity, including staff, funding, communication infrastructure and other resources, and learning from previous emergencies. Fourth, effective coordination is supported by high-level political leadership and incentives for collaboration. Country experience also highlighted interactions between these components, and with the wider health system and governance architecture, pointing to the need to consider coordination as part of a complex adaptive system.

**Conclusion:**

COVID-19 and other shocks have highlighted the importance of effective coordination and partnership across government and with other stakeholders. Using country experience, the paper identifies a set of recommendations to strengthen coordination for health system resilience and emergency management.

**Supplementary Information:**

The online version contains supplementary material available at 10.1186/s12913-022-08859-6.

## Introduction

COVID-19 has highlighted the importance of health system and wider governance for effective preparedness and response to shocks [1, 2]. Shocks can be defined as extreme stresses and challenges resulting from external events, including both acute crises such as floods or epidemic outbreaks, and slow-onset, protracted crises such as drought [3]. Shocks affect health system demand and supply, by changing the disease burden, access to and acceptability of services, and the health system’s capacity to meet population needs [4]. Effective governance is essential for managing both sudden and more protracted shocks [3]. This key role of governance is widely recognised in frameworks on emergency management and health system resilience, including frameworks from the World Health Organization (WHO), World Bank, and Universal Health Coverage (UHC) 2030 partnership [5–8].

Within discussions of effective governance, coordination and partnership are often identified as fundamental requirements. The need for effective coordination and partnership is indicated in frameworks on health systems broadly as well as frameworks focused on governance, in discussions of routine health system functioning and system strengthening as well as those on health system resilience and emergency management, and as important in humanitarian, fragile, and shock-prone settings [4, 6, 7, 9–15]. Indeed, a recent review of health system governance in settings with conflict-affected populations found that participation and coordination were the most frequently identified governance principles. Further, stakeholder collaboration was one of the most common facilitators of effective governance, while poor coordination, lack of a harmonized response, and lack of clarity on stakeholder responsibilities were among the most common barriers [16]. The need for stakeholder coordination and partnership is also emphasised in strategies, guidance and principles on emergency preparedness and response, disaster management and humanitarian response. For example, “country-level coordination, planning, and monitoring” is Pillar 1 in WHO’s COVID-19 Strategic Preparedness and Response Plan [17], and systems for national coordination were included in almost all countries’ Preparedness and Response Plans [18]. Similarly, Commitment 6 of the Sphere Handbook for Humanitarian Response requires that “communities and people affected by crisis receive coordinated, complementary assistance” [19].

While effective coordination and partnership are widely recognised as important, there is limited high-quality, practical evidence on effective mechanisms and approaches. A review of the effectiveness of coordination in humanitarian crisis found only four eligible studies, and highlighted the low quality of evidence [20]. Coordination and partnership tend to be considered as part of wider analyses of resilience or emergency management, which limits the depth of discussion; coordination and collaboration among different actors are noted as important, rather than unpacking specific approaches or enablers. This high-level approach reflects characteristics of the wider health system resilience literature, where a growing body of evidence has identified broad attributes for resilience, with less work to translate these high-level themes into specific, actionable steps [12]. Some checklists and assessment frameworks on emergency management provide more specific criteria related to coordination and partnership [21], but without the empirical evidence to illustrate and guide effective approaches. Recent work provides valuable examples to explain and assess coordination and partnership approaches in high-income countries [1], but there remains limited detailed examination of coordination and partnership for emergencies in low-income and fragile settings. Reflecting this, coordination and partnership across different actors for health system resilience have been identified as areas requiring further research [22, 23].

This paper aims to enhance understanding of key factors and approaches that enable effective coordination and partnership, in order to support efforts towards strengthening strengthen health systems resilience and emergency management. Drawing on empirical examples from experience with managing COVID-19 and other public health emergencies in a range of countries in Asia and Africa, the paper examines the role played by coordination and partnership with different stakeholders (such as NGOs, development partners, the private sector and local leaders, as well as government) in supporting health system preparedness and response to shocks; the strengths and weaknesses of government and stakeholder coordination structures at national and sub-national levels; and factors that have either enabled or hindered effective coordination. Based on this, the paper outlines key lessons for effective coordination structures and systems that can support preparedness and response.

Throughout the paper, we adopt a working definition of coordination as involving formal or informal mechanisms and arrangements for collaboration among stakeholder groups, designed to maximise the effectiveness and cohesiveness of action in support of emergency preparedness, management and response [24–26]. Partnership involves a collaborative relationship between two or more parties for the achievement of goals [27]. These definitions overlap, both involving collaboration, and coordination and partnership are closely linked. We see effective coordination as enabling effective partnership, providing a platform for different stakeholders to jointly work together, and effective partnership in turn supporting coordination, through collaborative relationships enabling agreement on cohesive and aligned approaches. Within this paper, we focus on coordination in terms of different stakeholders jointly planning and organising their activities to ensure they are aligned and cohesive, and partnership in terms of joint working between different stakeholders to share ideas and resources. Effective partnership has a number of wider dimensions, such as mutual respect and accountability [19], which are largely beyond the scope of this paper. Our focus in examining strengths, weaknesses, enabling factors and lessons rests primarily on coordination, with a view to ensuring that coordination can effectively enable stakeholder partnership. Given this focus, we primarily refer to coordination in the remainder of the paper. We also focus on coordination and partnership within national borders, rather than at transnational and global levels.

While our analysis considers preparedness for and response to shocks such as COVID-19, floods and drought, health system management of shocks overlaps with health systems resilience. Definitions of resilience vary, with some focused specifically on ability to prepare for, manage and learn from shocks [14], and others considering health system ability to manage a broader array of change and stress (as seen in discussions of ‘everyday resilience’ [28]. As shown in the discussion above, coordination and partnership have a central place in discussions of this broader concept of resilience as well as in relation to shocks.

## Methods

### Data sources

The study involved thematic synthesis [29] of 26 reports developed by or in collaboration with Oxford Policy Management (OPM), an international development consultancy that provides analysis and practical policy expertise to support low- and middle-income country governments and their partners in reducing poverty and disadvantage [30]. OPM is an affiliate of ReBUILD for Resilience, a research consortium that examines health systems in fragile and shock-prone settings to develop learning on ways to strengthen resilience, working with local and national governments and international health and development health agencies [31]. The reports included in the synthesis were developed as part of different OPM research, evaluation and technical assistance projects over 2019 to 2021, focusing on this time period due to the high volume of OPM work on resilience over these years. Reports were identified based on author knowledge of relevant OPM work, discussion with other OPM teams, and by searching internal databases and the organisational website. Reports were then selected for use in the analysis by the lead author based on provision of information about aspects of coordination and/or partnership in relation to management of health system shocks, including COVID-19. Coordination and partnership were the central focus for some reports, while others examined a broader range of issues but included information relevant for our research focus.

The reports used in the synthesis include formal research studies, rapid situation analyses, evaluations and other assessments, such as intra-action reviews undertaken in partnership with government. They are based on a range of methods, including primary data collection through interviews, focus groups and workshop discussions, analysis of secondary data, and other document review. Additional file 1 provides the full list of the original studies included in the synthesis, detailing the year, country, topic of focus, methods, authors and funder.

### Study settings

The reports included in the synthesis were based on primary data collection in 11 countries: Ethiopia, Kenya, Rwanda, Sierra Leone, South Africa, South Sudan, Uganda, Bangladesh, India, Nepal and Pakistan. Of these countries, Ethiopia and South Sudan are on the World Bank’s list of fragile settings for 2022, while Nepal and Sierra Leone have been included in this list in previous years [32, 33]. All 11 countries are shock-prone, with Rwanda and South Africa classified as at medium risk of humanitarian crises and disasters, and all others classified as at high or very high risk [34]. The range of shocks frequently experienced varies between countries, but includes floods, drought, displacement due to conflict or climate shocks, and disease outbreaks, and in some countries, earthquakes (for example, Nepal) or cyclones (for example, Bangladesh). In all countries, COVID-19 has added to other public health emergencies.

### Data analysis

Reports were synthesised using a coding framework to bring together information on similar issues (see Additional file 2). Some codes were identified deductively based on existing literature, the research questions and knowledge of the reports’ content, while others were added inductively during analysis. Codes related to aspects of national and subnational coordination, including structures, strengths, weaknesses and specific issues such as authority and clarity on roles, and to coordination and partnership with different actors (such as the private sector, local leaders and development partners), including their roles, mechanisms for and strengths and weaknesses of coordination with these actors.

Extracted data were compared and contrasted to search for patterns and differences across settings. Information and themes were then combined into core overarching elements, which were used to organise the synthesis write-up.

To support reliability of synthesis findings, we considered the strength of evidence in reports during analysis, in particular by only drawing on reported findings where sufficient supporting evidence was presented. For example, this meant placing more weight on interpretations and conclusions with a clear, logical thread and specific detail, and that were consistent with the evidence provided and supported by triangulation across data sources or participants [35]. Using this approach (rather than excluding reports on the basis of a pre-determined hierarchy of methods or other aspects of quality) is in line with the approach to quality assessment in realist synthesis, considering ‘nuggets’ of useful information, such as selected conclusions or findings, in reports that may otherwise have weaknesses [36].

## Results

Across countries and types of shock, effective coordination and partnership were important influences on shock preparedness and response, either as a supporting factor when coordination was strong, or a limiting factor when coordination was weak. Based on country examples of both effective coordination and gaps, our synthesis identified key issues for effective coordination and partnership related to i) the value of inclusive and representative coordination and partnership among different actors, ii) structural aspects of coordination bodies, iii) adequate capacities for coordination, including learning, and iv) political enablers. We consider each of these areas in turn.

### Inclusivity of coordination across levels and sectors

Country experience indicated the importance of inclusive coordination across government sectors and levels, and with partners outside government. This section looks first at coordination within government, and then at coordination with other stakeholders. We also consider inclusivity of coordination in relation to equitable representation of women.

#### Coordination across government sectors and levels

Shocks are often multisectoral, affecting a range of social outcomes, and requiring action by multiple government ministries, including health but also sectors such as social protection, water and sanitation, agriculture and finance. The need for cross-sector coordination within government was widely seen during the COVID-19 response, and several countries established coordination structures that brought together different ministries. For example, in Sierra Leone, the Ministry of Defence led on overall coordination, operations and logistics, the Ministry of Information and Communication led on risk communications, the Directorate of Science Technology and Innovation coordinated ICT and data management, and the Ministry of Welfare, in coordination with the Ministry of Health and Sanitation (MoHS) mental health division, led the psychosocial pillar and was also involved in scaling up social protection. During the initial response period, representatives from each pillar met daily at the National COVID-19 Emergency Response Centre [37]. This involvement of ministries beyond health helped to identify and, to varying extents, mitigate, the effects of public health containment measures on areas such as education and livelihoods. However, there were indications that coordination did not sufficiently balance input from different sectors: some stakeholders – particularly within the MoHS – felt that health sector representation was inadequate, resulting in insufficient technical input to the response [38].

Gaps in multi-sector coordination also affected the COVID-19 response in Ethiopia, contributing to delays in action by sectors beyond health and duplication of effort [39, 40]. Lack of coordination between response pillars and teams also reduced effectiveness of the response in specific areas; for example, teams leading on water, sanitation and hygiene and on infection prevention and control (IPC) had insufficient links to other teams (such as case management), which reduced attention to infection control as a cross-cutting issue [22].

Examples from other types of shocks also illustrated the importance of cross-sector government coordination for effective preparedness and response. In Pakistan, development of advance forecasts by the Meteorological Department provided a positive example of multisector coordination. These forecasts gave disaster agencies and the provincial Health Departments time and information to prepare for droughts, and helped Planning and Development and Finance departments to allocate relief budgets [41]. However, there have also been gaps in cross-sector engagement. For example, risk mapping and needs analysis has been incomplete due to a lack of coordination between the Disaster Management Authorities (that conduct this analysis), and departments responsible for food, health, population and social welfare [41]. Similarly, insufficient coordination has reduced the effectiveness of emergency supplies. For example, the Food Department provided wheat and rice during droughts, but a lack of communication with the Nutrition Department sometimes meant ration bags did not have appropriate contents for all age groups [41].

Insufficient cross-sector coordination has also hindered drought response in Kenya. Allocation of county emergency funds is determined through negotiations, and the health sector competes with other sectors. The financial decision makers have limited engagement in technical discussions on drought preparedness and response, and decisions are affected by political negotiations and political influence from external stakeholders such as private sector water trucking companies. Consequently, funding may be allocated to other priorities, such as food relief and WASH, leaving insufficient funding for health [42, 43].

As well as coordination across government sectors, the reports indicated the need for coordination between national and local government. Experience in some countries during COVID-19 showed how coordination between levels could support the response. For example, In Ethiopia, technical and financial support from national government helped to strengthen the functionality of subnational emergency coordination structures [44–47]. There were also positive examples in Sierra Leone: in Kono district, the Office of National Security representatives assigned from national government collaborated effectively with the District Health Management Team to share roles, ensuring that support was based on understanding district needs [48]. However, emergency response decision-making for COVID-19 and the allocation of resources was largely centralised in Freetown, potentially delaying responses at the district level [38]. Similarly in Kenya, there was limited integration of sub-national priorities into national COVID-19 response plans, and inadequate communication from national to county levels regarding processes for planning, funding, procurement and other aspects of the COVID-19 response [49]. Reports from Kenya also showed gaps in coordination between national and county government for other shocks, such as drought. For example, structures for reporting county disaster activities to the national Ministry of Health (MoH) were unclear, some roles were duplicated between levels, and there were tensions in the relationships. Insufficient collaboration contributed to delays in release of national government funding for response to drought or other emergencies [43].

Effective national-local coordination was affected by a range of issues, including capacity at each level, lack of coordination among central government departments, weaknesses in coordination fora, and political tensions. Several of these issues have affected coordination between government levels in Nepal. Ongoing decentralization has increased the need for strong coordination with local government. However, lack of coordination between national ministries and departments, such as agriculture and health, and a lack of clarity on the role of federal agencies in supporting subnational government, has hindered communication with local government, contributing, for example, to parallel instructions to local government from different national agencies [50]. Coordination between national and local governments in Nepal was further hindered by lack of regular fora, capacity gaps, and political tensions, issues highlighted in later sections.

In Ethiopia, coordination between national and local levels has been hindered by lack of clear and consistent communication channels. Disaster management structures vary between regions, and this contributes to variation in channels to share information with the federal government; for example, different regional bodies share information with a range of national line ministries, the National emergency coordination centre, or other disaster management task forces and councils. Regional staff are also sometimes unclear who they should communicate with at national level. As a result, there are multiple information channels, leading to misinformation and confusion among coordination agencies and partners [51].

#### Coordination with stakeholders outside government

Country experience illustrated the value of coordination and partnership with actors outside government, including community leaders and civil society, research organisations and the private sector, as well as development agencies (discussed below). Coordination with civil society and local leaders enabled their support for response efforts in several countries. For example, In Ethiopia, India, and Sierra Leone, local governments worked with civil society organisations and local leaders to disseminate messages on COVID-19 [44, 45, 48, 52]. Local leaders, civil society organisations and volunteers also provided other forms of support, for example, voluntary maintenance and installation of hospital beds and equipment in Ethiopia [39], monitoring district and international borders to support surveillance and compliance with travel bans in Kono, Sierra Leone and Lamwo, Uganda [48, 53], and making masks and running community kitchens in Kerala [52]. Collaboration with religious leaders was also an important influence on effective shock response. During the early stages of COVID-19, religious leaders in Bangladesh, Kenya and Uganda played positive roles such as disseminating information and closing mosques or churches to support physical distancing [49]. In Pakistan, the relationship between government and religious leaders was more difficult: despite attempts at coordination, religious leaders opposed physical distancing measures and large religious gatherings continued, contributing to the spread of infection [54].

The value of coordination with research institutes and universities was particularly noted in reports on the COVID-19 response in Ethiopia. A national COVID-19 research consortium was established, and Scientific Advisory Councils were formed at national level and in some regions [39, 44, 45, 55]. These fora supported the Federal Ministry of Health, Ethiopian Public Health Institute, and Regional Health Bureaus, providing advice, technical guidance, and operational research to guide the COVID-19 response. Research institutes also helped to scale up testing capacity and IPC supplies [39, 44, 45, 55]. However, government reviews of the COVID-19 response indicated that stronger coordination would have enhanced the role of universities, as lack of established research coordination platforms contributed to delays in identification and implementation of research priorities [39].

Effective coordination with the private sector also enabled shock response. During COVID-19, governments in Ethiopia and Kenya worked with private sector manufacturers to increase local production of medical supplies [39, 49], and media companies supported communication of COVID-19 information through free airtime or information hotlines in Ethiopia and Uganda [45, 49]. The contribution of private health providers was varied, partly due to gaps in coordination. Private hospitals provided additional (though for some, unaffordable) treatment capacity for the COVID-19 response (for example, in Ethiopia, Kenya, and Uganda) [44, 49], but in some cases gaps in coordination hindered an effective role. For example, in Pakistan, insufficient coordination of government communication to private hospitals caused confusion regarding whether they should stay open for service provision [49]. In Bangladesh, some private health providers stopped offering services during lockdowns, in part due to travel restrictions and difficulty in passing police checkpoints. Closer coordination between government and private providers could have encouraged continued private sector service delivery and helped to avoid undue scrutiny from the police [56].

#### Coordination with, by and among development agencies

Coordination with development agencies (including bilateral and multilateral donors, NGOs, and both humanitarian and development organisations) was indicated in many reports as important for effective shock preparedness and response. The contribution of development agencies was prominent across different shocks and settings, including COVID-19 in all countries considered in the synthesis [39, 49, 52, 53, 56, 57], drought and nutrition emergencies in Kenya [42, 43], and drought, floods, conflict-induced displacement and disease outbreaks in Ethiopia [58, 59]. The role of development agencies included provision of funding, technical expertise and direct in-kind assistance in areas such as planning, monitoring, logistics systems, supplies, infrastructure, communications and community engagement, health workers, and service delivery, as well as development of innovations [39, 43, 49, 53, 56–59].

However, weaknesses in development agency support were also widely documented such as delayed, short-term, unpredictable and insufficient funding; only supporting response rather than preparedness; duplication or gaps in geographic coverage; multiple parallel funding streams; and insufficient alignment with local priorities [39–43, 49, 57–60]. While not all weaknesses can be addressed through coordination, the reports suggested that strong coordination could support more effective development agency engagement. For example, in Ethiopia and Rwanda, effective national coordination with partners for the COVID-19 response enabled clear division of roles, mobilisation of resources, and sharing of international evidence [39, 57]. Where coordination with development agencies was insufficient, this reduced the value of their input. This was the case in Kenya and South Africa [49, 57], and in Somali region (Ethiopia), where gaps in communication and information sharing with the regional government and lack of development agency attendance at Emergency Operation Centre (EOC) meetings contributed to duplication of agency activities and resources [47]. Similar issues were seen with the response to drought and floods in Pakistan: at province level, NGOs and local organisations liaised directly with Provincial Disaster Management Authorities when designing relief programmes, but lack of wider multi-stakeholder consultation across agencies and government resulted in gaps and duplications in disaster response [41].

Effective coordination with development agencies depends partly on availability of sufficiently inclusive platforms for discussion between government and agencies (discussed below), but also on development agency approaches – including commitment to working through coordinated planning and funding structures, coordination among development agencies, and collaborative relationships. On the former, using agreed coordination structures can improve the alignment of development agency contributions, as shown by use of the cluster system in Ethiopia. The nutrition cluster has relatively strong government engagement and technical capacity, and coordinates the work of 4 UN agencies and 15 NGOs. However, the health cluster is weaker and less able to ensure agency coordination. In addition, agencies sometimes bypass the cluster system, and channel funds directly to project activities rather than via systems for harmonised donor funding, such as the Ethiopia Humanitarian Fund. This contributes to duplication in allocation of funding and uneven distribution of resources [59].

The value of coordination among development agencies for enabling communication with government is also shown by examples from Ethiopia. A multi-NGO rapid response mechanism was established to strengthen coordination between international and local NGOs. Bringing together NGOs in the consortium helped to streamline liaison with donors, local and federal government, and other stakeholders, through the cluster system and other fora. Regular coordination in turn enhanced collaboration and strengthened emergency response, for example through faster, more widespread and more efficient provision of relief [59]. Contrasting experience from Nepal showed how lack of coordination among development agencies could hinder communication with government, as the National Reconstruction Authority (NRA) struggled to coordinate with development agencies due to a high number of different agencies working on diverse issues and through different funding mechanisms [61]. NGOs, meanwhile, found coordinating with government difficult due to numerous agencies and individuals from local to national levels. suggested that coordination between government and development agencies is hindered by insufficient coordination within each group [61].

The importance of collaborative approaches, among development agencies and in their relationships with government, was highlighted by country experience during COVID-19. In Ethiopia and Rwanda, collegial relationships, involving good will and trust and avoiding “unhealthy competition” (quote from a development agency in Ethiopia), contributed to effective joint planning across government and agencies for the COVID-19 vaccine rollout, with aligned priorities and clear division of roles. [57]. However, good will became insufficient when the response was more complicated: in Rwanda, agencies reported that more formal systems to delineate roles and share information were needed as the volume and diversity of vaccine donations increased [57]. In contrast, development agencies in South Africa and South Sudan reported that competition among agencies weakened coordination for vaccine rollout. For example, in South Africa, some agencies began projects in new areas that already had established partners, bringing duplication in partner support [57].

#### Gender equity and representation of women in coordination structures

Shocks often disproportionately affect women and girls. Preparedness and response need to consider gender roles and the different needs of men, women, boys and girls, and coordination and decision making structures should include women and girls, as a right and to inform decisions [49]. The reports included in the synthesis provided limited information on consideration of gender in coordination systems. However, at an early stage in the COVID-19 response, there were indications of women being underrepresented in coordination structures: in Kenya, there were six women in the 21-person National Emergency Response Committee on COVID-19 (29%); in Pakistan there was one woman in the 13-person Emergency Core Committee (8%), and an analysis of four district-level COVID19 task forces in Uganda found that women constituted 22.5% of members on average, and that men held the most influential positions [49].

There were also indications of insufficient consideration of gender in coordination with civil society and local leaders for the COVID-19 response. In Sierra Leone, while the government in Kono worked effectively with traditional leaders, traditional women’s leaders (called Mammy Queens), were not represented in district decision making in Kono, and the chiefs who were represented were all male [48]. Similar lack of women’s involvement in stakeholder engagement was seen in Bangladesh, where women’s rights organisations reported being left out of local and national consultations on the COVID-19 response [49].

### Structural aspects of coordination mechanisms

Several characteristics related to the structure of coordination mechanisms affected the strength of coordination for emergency management, including the overall availability of coordination fora, clear roles and mandate and sufficient authority for coordination bodies, and the value of ongoing, standing structures that enable development of capacity and learning and action before and after emergencies. These characteristics are discussed in turn below.

#### Availability of coordination structures and regular meeting fora

A basic requirement for effective coordination is the availability of fora where different actors can come together to discuss activities and share information. Country experience indicated the importance of such fora for information sharing, resource mobilisation and joint planning between government sectors and levels and with other actors.

Experience from Ethiopia showed the contribution of coordination fora at national and subnational levels, and difficulties for emergency response when such fora are lacking or irregular. During COVID-19, the National Public Health Emergency Operations Centre (PHEOC) under the Ethiopian Public Health Institute (EPHI) and subnational PHEOCs at regional level provided a forum to agree plans, share information and coordinate resources [39, 44, 46, 60]. Coordination through the national and regional PHEOCs, and via task forces and regular development agency coordination meetings, supported contributions to areas such as risk communication and community engagement (RCCE), case management and quarantine [39, 44, 46]. This in turn enhanced speed and effectiveness of response activities [44, 46]. Where there were gaps in consistency or availability of coordination fora, this hindered the COVID-19 response. For example, at national level, joint planning and prioritisation across government departments were affected by interruption of the weekly coordination meetings between the Federal MoH and the EPHI PHEOC, and lack of a platform to synergise COVID-19 modelling outputs from different institutions contributed to uncoordinated modelling and forecasting exercises. These gaps hindered decision making and meant interventions were sometimes poorly targeted or introduced before plans were finalised [39]. At local level, absence of zonal or woreda level PHEOCs (or adequate alternative coordination fora) in Somali and Oromia hindered resource mobilisation and sharing of information between levels, and contributed to duplication of partner activities and gaps in the response [45, 47].

Experience in Kenya, India and Uganda also indicated the role of subnational coordination fora. In Kenya, a range of county government coordination structures were established after devolution, such as County Steering Groups, which include line ministries and development agencies, and Disaster Management Committees, which include the County Executive, senior county officials and humanitarian agencies. These structures have improved stakeholder engagement, cross-sector coordination amongst line ministries and with development agencies, sharing of early warning and other information, and clarity on roles [43]. Partnership between development or humanitarian agencies and county health officials through these structures contributed to faster response for the 2019 drought, for example by helping to maintain buffer stocks for nutrition commodities, redistribute nutrition supplies. and scale up integrated health outreach [43]. In India and Uganda, subnational coordination fora also supported the contribution of different stakeholders in emergency response, this time with COVID-19. In Kerala, ‘intersectoral convergence meetings’ at district and block levels were used to outline roles and identify potential contributions from different actors, such as use of hotels for quarantine facilities [52]. In Lamwo, Uganda, the district COVID-19 taskforce provided a channel to request partner support and contributed to assistance in areas such as transport for referrals [53].

The value of functioning fora for securing and coordinating development agency support shown by the examples from Ethiopia, Kenya and Uganda was also evident in contrasting country experience with COVID-19 vaccine rollout [57]. In Ethiopia, Rwanda and South Sudan, regular meetings between government and development agencies helped to move activities forward, share information, establish relationships, clarify roles and avoid duplication in development agency activities, and provided a forum for development agency input to government plans. For example, in Rwanda, structures such as the interagency coordination committee, COVID-19 task force and technical working groups (TWG) included relevant government and development agency stakeholders and allowed joint planning and sharing of information. In contrast, South Africa did not have a clear or consistent forum for coordination between government and development agencies on COVID-19 vaccine rollout. Lack of coordination among UN agencies is a longstanding and wider challenge in South Africa, partly related to absence of an interagency coordination committee for immunisation. There was some development agency engagement in high-level national committees such as the National Advisory Group on Immunisation, but these high-level fora were restricted to senior officials and overall policy direction, so did not provide an opportunity for discussion among technical staff to coordinate implementation. With many development agencies working on COVID-19, the absence of regular coordination fora brought gaps in information sharing and joint planning, and consequent duplication of roles, confusion around different tools and approaches for vaccine rollout (for example, with different agencies introducing their own monitoring and reporting systems), and missed opportunities for greater impact through aligned and pooled resources. Absence of fora for joint discussion among development agencies and government also increased pressure on government staff time, due to multiple meetings with individual agencies rather than one joint fora [57]. A similar gap in fora for joint discussion between development agencies and government was seen in Kenya for the broader COVID-19 response. Decision making was led by a central body only accessible to top government officials and with no development agency involvement (the National Emergency Response Committee), contributing to insufficient coordination between government and agencies on areas such as provision of laboratory supplies [62].

As well as supporting coordination between government and other stakeholders, regular fora supported collaboration between government levels. This role was shown by use of subnational EOCs in Ethiopia for earlier emergencies. In 2018, the Federal Government established EOCs in two zones affected by an increase in conflict-induced displacement (Gedeo and West Guji), the first time subnational EOCs had been used. These EOCs convened federal NDRMC representatives, zonal and woreda officials, and despite difficulties related to their new development, the EOCs helped to ensure federal decision makers understood local issues and to support coordination between federal and local government (as well as generating real-time information and supporting coordination with humanitarian agencies). This coordination in turn supported effective partnership in response and rehabilitation activities, such as targeting and verifying food aid distribution [40]. In contrast, experience in Nepal showed how inadequate meeting fora could hinder coordination between government levels: the Disaster Risk Reduction and Management National Council provided a potential forum for coordination between government levels, as provincial Chief Ministers are members. However, meetings were ad hoc and tended not to discuss inter-governmental coordination, contributing to coordination gaps [50].

#### Clear roles, mandates and sufficient authority for coordination bodies

A second structural aspect identified in our analysis is the need for coordination bodies to have clear roles and adequate mandates and authority. There were several examples of multiple bodies being involved in coordination, with insufficient clarity on their roles and overlapping mandates hindering the response.

In some cases, overlapping mandates resulted partly from development of new structures, sometimes in response to emergencies. In Pakistan, after the 2005 Kashmir earthquake, the government established the Earthquake Reconstruction and Rehabilitation Authority (ERRA) to support rescue, relief and reconstruction in the affected regions. In 2007, a National Disaster Management Authority (NDMA) was established. In 2011, the government decided to merge the ERRA into the NDMA, but with the ERRA continuing its on-going projects until 2019. Creation of the NDMA and continuation of the ERRA brought overlap and confusion regarding the roles of these two institutions. This contributed to slowing the reconstruction effort through longer and more complex policy, bureaucratic and financial management processes [61]. There have been further overlaps with the roles of the Natural Disaster Management Commission, Provincial Disaster Management Commissions, and Provincial and District Disaster Management Authorities, bringing ambiguities regarding responsibilities between different bodies and government tiers [63].

Insufficient clarity on the roles of different coordinating organisations and teams also hindered emergency management in Ethiopia. In particular, ambiguity in division of roles for nutrition emergency management between the national MoH, EPHI, and NDRMC have hindered development of coordinated structures for nutrition emergencies at subnational levels [59]. Overlapping structures and uncertain roles also affected the COVID-19 response. For example, coordinating teams linked to the Ministry of Peace, MoH, EPHI, NDRMC as well as city administrations and civic associations were all involved in resource mobilisation. Insufficient clarity on roles or coordination among these bodies led to fragmentation and duplication in resource mobilisation, for example with similar requests being submitted by different agencies to the same donor, lack of shared and complete information on the quantity or type of resources donated, and difficulties in resource allocation and distribution [39]. There were also overlaps in the roles of teams coordinating different response pillars (e.g. case management, IPC, surveillance, and RCCE), leading to duplication of effort [39].

Experience from Ethiopia also provides examples of effective work to clarify the roles of different organisations. With the 2017–18 IDP crisis in Ethiopia, an accountability matrix was developed for the IDP camps detailing who was responsible for what, and each sector and cluster was responsible for activities related to its mandate and expertise [40].

As well as avoiding overlapping mandates, coordination bodies need a sufficiently wide mandate for all relevant shocks. In Kenya, the new county institutions such as Disaster Management Committees and Disaster and Humanitarian Coordination Directorates are designed to support a multi-hazard response. However, the National Drought Management Authority (NDMA) tends to be stronger and so often provides de facto leadership for county coordination bodies. Shocks such as floods and disease outbreaks fall outside the NDMA’s official mandate, contributing to a focus on droughts in county disaster management plans and gaps in preparedness for other shocks [43].

Sufficient authority for coordinating bodies is also important, affecting their ability to convene other agencies and ensure effective response. As above, the Kenya NDMA has played a leading role in coordination. However, the NDMA has no authority over the county line ministries responsible for implementing disaster management and response plans, reducing implementation of agreed actions [43].

Authority has also influenced the effectiveness of coordination bodies in Ethiopia. The position of the National Disaster Risk Management Commission (NDRMC) within government structures has changed over time, affecting the NDRMC’s ability to effectively coordinate emergency response [40, 60]. The NDRMC was originally established in 2015 as an autonomous body under the Office of the Prime Minister, with a remit to coordinate emergency response, and mainstream disaster risk management across government. This autonomous position gave the NDRMC increased status compared to its predecessor (the Disaster Risk Management Food Security Sector, DRMFSS), which was under the Ministry of Agriculture, and consequently strengthened its convening and coordinating authority. Together with support from humanitarian partners, autonomous status helped to improve the response to the 2015–16 El Niño drought. After 2018, the NDRMC was absorbed under the new Ministry of Peace. As a department within a ministry, the NDRMC could only promote its policy objectives through the Minister, and had only an indirect relationship with the higher level National Disaster Risk Management Council chaired by the Deputy Prime Minister. The new structure also meant the NDRMC was not regarded by other ministries as an equal partner. This reduced the NDRMC’s coordinating authority with line ministries, which in turn hindered its capacity to promote disaster risk management mainstreaming, and affected coordination for preparedness and response. For example, insufficient authority over line ministries made it harder for the NDRMC to access and collect early warning data [40, 60]. Similar structural issues have affected the authority of coordinating bodies in Nepal. The NRA is a separate institution with frequent and direct access to the Prime Minister and the Council of Ministers. There have been challenges in coordination between the NRA and other ministries, but Prime Ministerial oversight of NRA management meetings helped flow of funds and provided high level support for decisions, and the NRA’s relative authority supported collaboration across ministries to ensure effective response [61].

#### Use of existing coordination structures and ongoing coordination before and after shocks

Use of pre-existing coordination structures to support shock response enabled coordination in some countries, by providing established systems and capacity, relationships and ways of working. This was seen with support for the COVID-19 vaccine rollout [57]. For example, in Ethiopia, several committees and working groups used for COVID-19 coordination were part of routine immunisation programme structures, including the National Immunization Technical Advisory Group and TWGs on supply and logistics and communications. The same organisations and individuals who were part of these routine structures were involved for COVID-19 vaccination, so using these structures was more efficient, and their established strength supported COVID-19 coordination. Using routine immunisation structures also helped early clarity and agreement on roles, as development agencies and government had established focus areas for routine immunisation (such as communications or supply chains), and retained these roles for COVID-19 vaccine rollout. For the broader COVID-19 response, existing platforms for coordinating development agency resources helped effective identification, mobilisation and allocation of funding. Similarly, using the existing PHEOC structure, previously activated for Ebola, enabled rapid PHEOC activation for coordination during COVID-19 [39]. In contrast, newly created structures may need additional support to build capacity. In Ethiopia, some new TWGs created for COVID-19 vaccine rollout (for example, on planning, monitoring and evaluation) were weaker, for example meeting less frequently, partly due to lack of established government capacity to engage [57].

Country experience also shows the need for coordination structures to function on an ongoing basis, before and after shocks. In several countries, coordination structures only became active during emergencies, slowing response and limiting action on preparedness and recovery. In Kenya, County Steering Groups for disaster management were supposed to meet monthly, but meetings were irregular outside emergencies and only took place when there was a drought. This delayed discussion of early warning bulletins and limited coordination to response rather than the anticipatory planning needed given increased regularity of climate shocks [43]. Similarly in Pakistan, cross-sector collaboration between government departments and development agencies was limited to emergency situations rather than pro-active advance planning [41], and in Ethiopia, the high level national Disaster Risk Management Council and sectoral task forces or committees have tended to meet only when an emergency arises [40, 59]. This lack of a permanent, ongoing structure has delayed response as committees need to be activated at the time of emergency, and limited recovery capacity [40, 59].

### Adequate capacity of coordination bodies and learning from previous shocks

Country experience showed the influence of organisational capacity on coordination, including technical and political skills, sufficient staff, infrastructure and funding.

The influence of human resource capacity in national coordination bodies was seen in several countries. In Nepal, NRA staff were seconded from line ministries and all positions were temporary. This secondment structure brought several difficulties that reduced human resource capacity: high turnover, which affected institutional memory; a lack of coherence and complementarity in skills; lack of rewarding career paths within the NRA to attract high calibre staff; and hierarchical issues related to individuals having less senior positions in the NRA decision making hierarchy compared to in their parent ministry, with consequent demotion. Previous experience in Nepal also indicated the importance of political skills among the leaders of coordination bodies, and sufficient credibility of these leaders with the wider government bureaucracy, to ensure they can win support and cooperation from staff and other ministries [61]. A further consideration from experience in Nepal was flexibility of institutional capacity, with a permanent core structure that can expand as needed for disaster response, including through additional staffing [61].

Further human resource challenges for coordination bodies were indicated by experience at the NDRMC in Ethiopia. One issue was the overall availability of skilled staff: the Commission had skilled technical staff, but some experienced staff were leaving, and there were insufficient skilled personnel to provide technical support on disaster management mainstreaming to line ministries or to monitor policy implementation. NDRMC staff capacity to support coordination was also strained by multiple concurrent emergencies; for example. early NDRMC engagement in the COVID-19 response was limited by simultaneous work on a desert locust plague, major floods, and conflict-induced displacement [40, 60]. Commission experience also highlighted the need for specific technical skills to manage different types of shock: the NDRMC’s historical strength has been with drought response, but a changing humanitarian environment, particularly increased conflict and displacement and more recently COVID-19, required new skills [40].

Country experience also showed the influence of human resource capacity on subnational coordination. In Nepal, local governments were relatively newly established and still building organisational capacity, including for disaster management. Many local authorities had established or begun developing EOCs, but a lack of human resources contributed to limited EOC functionality and ability to ensure effective coordination [50]. Elsewhere, the influence of human resource capacities on subnational coordination was evident in relation to COVID-19. In Pakistan, insufficient technical expertise for emergency preparedness, response and coordination reduced the capacity of provincial coordination bodies [41]. In Ethiopia, subnational capacity varied between regions and levels. For example, in Oromia, availability of trained staff assigned to emergency response at regional level supported early activation of the EOC, communication across levels, and development of plans to support coordination. However, insufficient staffing and experience at zone and woreda levels contributed to lack of sub-regional EOCs and hindered coordination [45]. In Sidama, a new regional administration, limited training meant insufficient technical skills and experience in emergency coordination at regional level, and there were also insufficient dedicated staff at sub-regional levels. This reduced the functionality of regional and woreda Emergency Coordination task forces and EOCs for the COVID-19 response, hindered coordination, and contributed to delays and insufficient harmonisation of response activities [44]. Insufficient skilled staff also hindered coordination between federal and subnational government [39, 51].

Experience with subnational coordination capacity for the COVID-19 vaccine rollout in Ethiopia highlighted the influence of ongoing health systems human resource issues: long-standing shortages of government and immunization programme staff contributed to limited time capacity to participate in coordination fora, which reduced the functionality of some regional and zonal working groups for COVID-19 vaccination. Weak functioning of subnational working groups in turn reduced their capacity to coordinate with national structures, delayed sharing of information, and hindered vaccine rollout in some regions [57].

As well as staff, subnational capacity was affected by funding and communications infrastructure. For example, in Pakistan, availability of accurate and consistent information to support coordinated action has been limited by insufficient technology and connectivity at lower health system levels, as well as by insufficient administrative capacity, unfamiliarity with information systems, and incentives related to self-reporting [41]. In Nepal, provincial EOC capacity is limited by shortages of equipment and funding, as well as staffing [50]. In Ethiopia, phone outages and subnational gaps in internet access and information technology hindered emergency coordination and information sharing between regional, woreda and federal government and with regional development agencies, including during COVID-19 [51]. Where communications equipment was available, for example in the Southern Nations Nationalities and People's Region and Oromia, this enabled information sharing and coordination [45, 46]. For example, in Oromia weekly virtual meetings with leadership in all zones and towns helped to identify supply gaps and distribution needs [45]. In South Sudan, unreliable internet was further compounded by disruption to road networks, limiting sub-regional attendance at physical meetings to discuss the COVID-19 response [57].

Adequate funding also affected capacity for coordination, partly due to allowances associated with participation in coordination meetings. In Kenya, strained county health budgets hinder coordination mechanisms, for example when nutrition coordination meetings cannot be hosted due to lack of funds [43]. Lack of budget has also affected development agency coordination with local government for the COVID-19 response in South Sudan, due to the costs associated with workshops [57].

Capacity is needed not just within the health sector, but across sectors given the importance of multi-sector coordination and action. In Ethiopia, experience during COVID-19 showed that several government ministries and departments lacked staff with training and experience in public health emergency management and coordination, and did not have established structures to support disaster management. This contributed to difficulties in cross-sector coordination for the COVID-19 response, and increased reliance on the health sector [39].

Development agencies can support the capacity of coordinating structures; for example, they supported communications infrastructure in some regions of Ethiopia [51]. However, short term support has limited the effectiveness of capacity development. In Ethiopia, development agency support to the NDRMC has often involved short-term technical assistance. This temporarily boosted capacity, but technical assistance staff sometimes lacked personal investment in the NDRMC, and their short terms of appointment hindered sustainable improvement and institutional learning [40]. Similar sustainability issues were seen in Pakistan, where development agencies provided capacity building for the Province Disaster Management Agencies and Health Departments, but with insufficient ownership and gaps after donor funding ended [41].

#### Using previous learning to support effective coordination

Country experience showed that learning from previous shocks could support effective coordination, by demonstrating the value of partnership and so encouraging coordination efforts, and by developing mechanisms for coordination or lessons on effective coordination approaches.

In Kerala, experience with a series of shocks (such as floods and the 2018 Nipah virus outbreak) showed government the value of citizen engagement, and helped it to develop mechanisms to convene other government departments and to collaborate with external stakeholders. This in turn supported coordination for the COVID-19 response [52]. In Sierra Leone, learning from Ebola contributed to active community engagement and involvement of traditional leaders in the COVID-19 response, and enabled swift activation of coordination structures and systems for COVID-19, such as use of the Ebola Emergency Operations Committee [37, 38]. Similarly, previous experience of emergency coordination using an incident management system in some regions of Ethiopia facilitated establishment of the regional PHEOC and task forces for COVID-19 coordination, which in turn supported engagement with partners and other stakeholders [44, 46]. Regional governments have also used learning from past droughts to support coordination during nutrition emergencies, including holding more frequent coordination meetings and creating different sector task forces [40].

While learning can support effective coordination, country experience also indicated several factors that can limit identification and use of lessons from previous emergencies. In Ethiopia, the NDRMC had used lessons from previous years to strengthen coordination of drought relief, but more extensive learning was limited by issues such as lack of opportunity to reflect as crisis rapidly follow one another; limited opportunity to absorb lessons from evaluations or reviews; coordination structures often becoming dormant after emergency response is concluded; and a lack of leadership to act on learning [40]. The latter issues also hindered learning in line ministries: task forces and committee meetings only took place during emergency response, reducing opportunities to discuss and reflect on learning after crises end [59]. In addition, sector focal points were temporarily assigned for emergencies and then returned to their usual duties, and they were not directly accountable and evaluated for their involvement in emergency management [40], hindering incentives and opportunities for learning. At subnational level, staff turnover and poor documentation were further issues hindering learning for development of effective coordination systems [40].

### Political considerations and effective government leadership

The final area identified in our analysis was the role of political considerations, including leadership across different government levels, sectors and stakeholders, and incentives for coordination.

Experience during COVID-19 indicated the value of national government leadership for coordination between government sectors and levels, and with development agencies. In Rwanda, government led coordination for vaccine rollout, and asked development agencies for assistance in specific areas, which helped to harmonise their support [57]. In Pakistan, the Prime Minister’s political ownership of the National Command Operation Centre (NCOC) established for COVID-19 supported the NCOC’s effectiveness and contributed to engagement of other government sectors and departments [41]. Similarly in Ethiopia, the National Ministerial Committee established for COVID-19 was accountable to the Prime Minister, the Minister of Health chaired the overall COVID-19 coordination group, and senior government leadership provided close follow-up and support for activities [39, 57]. This high-level leadership and engagement promoted collaboration across different national government ministries, and coordination with development agencies and subnational levels, including through early activation of the PHEOC and creation of multi-stakeholder coordination fora [39]. High-level national leadership also supported coordination at subnational levels, particularly via material and political support for the functionality of regional PHEOCs [44–47].

Leadership at subnational levels also affected the strength of coordination during COVID-19. In Pakistan, effective leadership by Chief Ministers in some provinces contributed to cross-sector coordination, such as involvement of the district administration, police, and government departments on health, education, water, sanitation and others in quarantine facilities [41]. In Kerala, leadership from the Chief Minister to establish a platform for discussion across ministries helped to ensure engagement of other state ministries, with all departments (not just the Ministry of Health) instructed by senior government to focus on COVID-19 [52]. In Ethiopia, where regional political leaders and senior regional government were actively engaged, this facilitated early establishment of the EOC, availability of staffing for emergency coordination platforms, coordination across sectors, links between the regional EOC and lower levels, and engagement with stakeholders such as health professional associations [44, 45] Where political leaders were less engaged, this made information sharing, communication and coordination of the response less systematic or streamlined [39, 45, 46]. For example, in Oromia, political engagement declined as growing political instability took leaders’ attention. The resulting leadership gap contributed to reduced engagement by departments beyond health, interruption of coordination fora, insufficient resources for the response, and duplication of some activities [45].

While high-level political leadership supported engagement of different government sectors, effective multi-sector coordination also required sufficient leadership within different government sectors, including ministries beyond health. In Ethiopia, national policy designates lead institutions to implement disaster risk management for different hazards; for example, the Ministry of Agriculture for agriculture-related emergency management, the Ministry of Water, Irrigation and Environment for floods, the Ministry of Peace for conflict-related emergencies, and the MoH for health-related emergencies. However, commitment to disaster management from ministers in other sectors has been limited, particularly beyond the MoH, with a tendency to rely on the NDRMC secretariat. This lack of leadership contributed to limited development of sector disaster management plans and structures, which in turn reduced of focus on and capacity for disaster management, and hindered coordination. For example, lack of clear responsibility and structure in other ministries weakened effective participation in multi-sector coordination fora and accountability for action [60].

Experience during COVID-19 indicated the need to balance high-level political or government leadership with sufficient technical input, from within and outside government. In Ethiopia, health professional associations, development agencies, technical government staff and other technical experts were part of coordination structures [39]. For example, coordination of vaccine rollout was initially managed by MoH, but government later brought in the wider, existing immunisation structures to provide technical input (e.g. the National Immunization Technical Advisory Group and the immunisation TWG) [57]. In contrast, in South Sudan, senior government officials decided to change the vaccine distribution strategy previously agreed by government and development agencies, without consultation. This led to delays in implementation, reduced population access, and ultimately to vaccine doses being unused and returned to COVAX. A later change in government structures brought more collaboration with development agencies and use of technical guidance, leading to a revised and more effective distribution strategy [57]. A final example from Sierra Leone showed the importance of balancing political leadership with engagement of technical government staff: MoHS staff were concerned that decisions made by political leaders with insufficient input from technical and medical experts reduced the effectiveness of COVID-19 response strategies [38].

Beyond leadership, other political considerations were also evident in country experience. A particular issue was political tension between national and subnational government, which hindered coordination across levels. In Pakistan, there was friction between national and provincial governments in the COVID-19 response, with open disagreement regarding some provincial policies (such as a more complete lockdown in Sindh). This tension partly reflected wider political systems, with different political parties in government in different provinces and at federal level [54]. In Nepal, the District Disaster Management Committee could support national-local coordination as the committee chair is a federal government official (the Chief District Officer, or CDO), and members are politically-elected local government leaders. However, the Committee’s effectiveness is reduced by concern among local leaders that CDOs exert central government power and side-line subnational government [50].

A further political consideration indicated by country experience related to incentives and openness regarding information sharing. Country experience showed that timely, open and accurate information sharing and unified data systems can support coordinated action, as well as underpinning other key elements of shock response such as identification of affected populations [41, 61]. However, political incentives sometimes reduced availability of accurate data. In Ethiopia, early warning information underpins the annual Humanitarian Requirements Document and Response Plan (the basis for government and agency work and for coordinated action). However, early warning information is collected by different ministries and government agencies, and sometimes withheld or manipulated, partly because this early warning data plays a role in determining budgets and resources. For example, tension over early warning information between the NDRMC and Ministry of Agriculture (which manages the Productive Safety Net Programme, PSNP) reflected concern about the size of the PSNP caseload and affordability of safety nets. Resources to the regions are also affected by population size, and associated under- or over-estimates of population figures reduce the accuracy of needs assessments. Political concerns over a perception of Ethiopia as an aid dependent ‘famine country’ have also led to late publication of early warning findings. One example came from the 2017–19 Gedeo/West Guji displacement crisis, where IDP numbers were sometimes inflated to attract additional resources, or understated to protect political image. These tensions reduced the effectiveness of early warning information, and both reflected and contributed to weaknesses in coordination [40]. Similar issues have affected more recent NGO response to nutrition emergencies:, gaps and delays in provision of information from subregional to regional level, delays in reporting of crises from regional government to the federal level and other stakeholders, and discrepancies between woreda and regional data regarding the number of people in need meant early warning information was late and uncertain, which hindered timely and coordinated NGO response [58].

The role of transparency in information sharing was seen with COVID-19 vaccine rollout in Rwanda and South Africa. In both countries, there have been gaps in information from government, including difficulty in accessing health management information system data in Rwanda (for example, on coverage of priority groups), lack of budgetary information on resources provided and funding gaps in both countries, and in South Africa, limited information on vaccine purchasing agreements. This lack of shared information on progress, costs, and resources hindered effective coordination and prioritisation of development agency support [57].

### Discussion

This paper has highlighted the importance of effective coordination and partnership for emergency preparedness and response in fragile and shock-prone settings. Based on empirical examples, the paper identified a set of issues that characterise and enable effective coordination. Overarching issues involve inclusive coordination across government sectors and levels and with other stakeholders, such as development agencies, NGOs, the private sector and research institutes; structural issues, including the availability of coordination fora, ongoing coordination structures that function before and after shocks, the mandate and authority of coordinating bodies, and streamlined information systems; the capacity of organisations with a role in coordination, including staff, skills and infrastructure, across relevant sectors and at different geographic levels; and political considerations and incentives, including government leadership.

The synthesis findings suggest key elements that can enable effective coordination, both for preparedness and response to shocks. Table 1 summarises key findings and recommendations.

**Table 1:** Summary of findings on key elements of effective coordination

Domains of effective coordination	Key elements and practices
Inclusivity of coordination	• Engagement and input from all relevant government sectors (multisectoral coordination), including sufficient technical input on health but also coordination with areas such as water and sanitation, social protection, agriculture, disaster management, and finance • Effective cooperation and communication between national and local governments (vertical coordination), including consistent and aligned guidance to local levels, clarity on roles, clear systems for reporting information upwards, and national government responsiveness to district needs • Government engagement with other stakeholders, including development agencies, local leaders and civil society, religious leaders, research institutions and the private sector • Development and humanitarian agencies working through agreed coordinating structures and funding systems, and approaching work collaboratively • Coordination and unified contact points among development agencies on the one side, and among government stakeholders on the other, to facilitate communication between multiple development agencies and government bodies • Gender equity in representation and involvement in coordination fora, across government and other stakeholders
Structural features of coordination mechanisms	• Availability of functioning coordination structures, including regular meetings at national and sub-national levels • Clear roles for each coordination structure and organisation responsible for coordination, including at different government levels, to avoid overlapping remits. Tools such as accountability matrices can help to avoid duplication and clarify responsibilities • Clear relationships and sets of responsibilities between disaster risk management coordination authorities and sectoral ministries • Mandates for coordination bodies that are sufficiently wide to support responses to the range of relevant shocks • Sufficient authority for coordination bodies to convene relevant actors and ensure the implementation of agreed plans. Positioning directly under the president or prime minister (rather than in a ministry) can support this authority • Coordination structures that function on an ongoing basis—before shocks occur to enable preparedness and anticipatory planning, and after shocks to support learning and recovery • Using existing structures can support coordination during shocks, by providing established organisational arrangements, roles, relationships and ways of working; new structures may require additional support
Adequate capacity of coordination bodies and use of learning from previous shocks	• Adequate numbers of staff with political and technical expertise, including expertise for all relevant types of shock, within coordination bodies at different levels • Career paths that reward expertise and provide stability of employment for at least core staff, to support skills, motivation and accountability • Adequate funding and communications infrastructure for organisations responsible for coordination, across relevant government sectors and levels • Support for sustained and ongoing capacity, rather than short term technical assistance or capacity only during emergencies • Learning from previous shocks, enabled by time for reflection, leadership to act on learning, accountability for emergency response as part of core staff roles, and the retention and exchange of organisational learning, including through standing (rather than ad hoc) coordination structures
Political considerations and effective government leadership	• Effective political leadership, at national and subnational levels and in different government sectors, balanced with technical input from government and other stakeholders • Political, organisational, and individual incentives to support coordination, including in relation to transparent and accurate information sharing • Regular communication and reporting across government levels to promote effective subnational leadership and accountability

The synthesis demonstrates the complexities of coordination, resulting from issues such as myriad actors, requirements for multiple capacities and insufficient resources, power imbalances, the influence of pre-existing health system conditions, and the role of factors both within the health system and beyond, such as wider governance, national infrastructure and support from other sectors. The findings also demonstrate the interactions between different components and feedback loops, for example with human resource systems affecting capacity to learn from previous shocks, and scope for learning then affecting availability of adequate expertise. Similarly, high-level political support can affect the authority of coordinating bodies, in turn influencing engagement and information sharing by different sectors. These interactions reinforce the emphasis on systems approaches in the health system strengthening and resilience literature [64, 65].

Our findings build on existing analyses and strategies that emphasise the importance of coordination and partnership across sectors, levels and stakeholders. Some specific enablers and characteristics of effective coordination identified in our synthesis have also been identified in other contexts. For example, a recent discussion of experience with coordination during COVID-19 indicated the importance of responsive leadership and strong political will, clear roles and responsibilities, and effective information systems, as well as the need for coordination across sectors, levels, and with community and religious leaders [66]. Weak leadership, shown in our findings as affecting engagement from different government sectors and levels, has also been identified as hindering coordination for shock response in other reviews [14, 67]. In humanitarian contexts, the role of collaborative relationships, development agency commitment to alignment, capacity for coordination, and effective information management have been identified as enabling coordination [68].

Some characteristics and enablers identified in our synthesis are also stipulated in existing guidance on coordination for emergency management. In particular, the WHO toolkit for assessing crisis management capacity indicates the need for a high-level multisectoral committee with regular meetings, a clearly defined mandate and set of responsibilities, clear authority, and sufficient staff, equipment, funding and support systems to fulfil its mandate [21], in line with our findings on the structural requirements for coordination bodies and the influence of capacity on coordination. This toolkit also emphasises the need for coordination across government sectors and with the private sector and civil society.

Several other characteristics and enablers of effective coordination from our findings have been discussed as key requirements for broader shock response, health systems resilience or health systems strengthening, rather than in direct connection to coordination and partnership. For example, the roles of political leadership, equitable gender representation, functioning information systems, learning, and technical skills and staffing for emergency management are widely highlighted as important for broader health system strengthening, emergency management and resilience [69–72]. Our work shows the specific relationship of these requirements to effective coordination for shock preparedness and response, illustrating interactions between coordination and other key health systems and resilience capacities.

This analysis was restricted to information from reports developed or supported by OPM, and therefore did not aim to provide a comprehensive assessment of all aspects of coordination and partnership, or a full review of available evidence. Synthesising findings from OPM reports brings new evidence to the discussion, as some reports were not publicly available, and none had been published in the academic literature, but a wider review of all available research would provide additional evidence. The reports included in this review had different areas of focus and research questions, and as such, they did not provide directly comparable information across countries. Due to varied focus and purpose of the original reports, some provide in-depth discussion and detail, whereas others provided only summary information on coordination and partnership. Reports were not evenly spread across countries; for example, many reports were from Ethiopia due to a substantial OPM programme on resilience. However, the reports did cover varied country contexts, which helped to identify variations in the strength and approach to coordination.

### Conclusion

COVID-19 and other shocks have highlighted the importance of effective coordination and partnership across government and with other stakeholders. We identified four key areas that characterise and enable effective coordination for emergency management, involving inclusive approaches, and the structures, capacities, and political incentives for coordination. Within this, the synthesis identifies a set of issues to consider in strengthening coordination for health systems resilience and shock response, such as political skills, organisational authority, retention of learning, communication platforms, and alignment by development agencies. Interactions between these components and with the wider health system and governance architecture indicate the need to consider coordination as part of a complex adaptive system, so requiring attention to interdependencies between different components.

The paper raises several areas where further evidence could support understanding of effective coordination systems. These include the political economy of coordination, including the roles and membership of coordination structures and incentives for engagement among different ministries; effective structures and formats for coordination fora, such as optimal constitution and size; gender equity in coordination, including representation of women in decision making structures, and strategies for coordination that have supported an inclusive response; the influence of funding systems on coordination, including use of pooled funding; and variation in coordination between different types of shock, including acute or protracted crises and whether shocks are framed as ‘health’ emergencies, and how this affects representation of health and other sectors.

## Supplementary Information

**Additional file 1.** Reports and projects providing evidence for the synthesis.

**Additional file 2.** Coding framework.

## Data Availability

This paper is a synthesis of project reports and does not analyse primary data. As such, data sharing is not applicable to this article as no datasets were generated or analysed during the current study. Ethics approval and consent to participate This paper synthesises evidence from other research reports. As such, ethics approval was not required.

## Abbreviations

BMGF: Bill & Melinda Gates Foundation
CDO: Chief District Officer
CMAM: Community-based Management of Acute Malnutrition
EOC: Emergency Operations Centres
EPHI: Ethiopian Public Health Institute
ERRA: Earthquake Reconstruction and Rehabilitation Authority (Pakistan)
FCDO: Foreign, Commonwealth and Development Office
FMOH: Federal Ministry of Health (Ethiopia)
IPC: Infection prevention and control
NCOC: National Command Operation Centre (Pakistan)
NDMA: National Disaster Management Authority (Pakistan)
NDMA: National Drought Management Authority (Kenya)
NDRMC: National Disaster Risk Management Commission (Ethiopia)
NGOs: Non-governmental organisations
NRA: National Reconstruction Authority (Nepal)
OPM: Oxford Policy Management
PHEOC: Public Health Emergency Operations Centre
PSNP: Productive Safety Net Programme
RCCE: Risk communication and community engagement
SNNPR: Southern Nations Nationalities and People’s Region
TWG: Technical Working Group
UHC: Universal Health Coverage
UNICE: United Nations Children’s Fund
WHO: World Health Organization
