# Supplementary material for: How can we strengthen partnership and coordination for health system emergency preparedness and response? Findings from a synthesis of experience across countries facing shocks
Source: BMC Health Serv Res. 2022 Nov 29;22:1441. doi: 10.1186/s12913-022-08859-6 (PMC9706990; doi:10.1186/s12913-022-08859-6)
Supplement: Supplementary file 1 — Additional file 1. Reports and projects providing evidence for the synthesis. [file 12913_2022_8859_MOESM1_ESM.docx]

Additional file 1: Reports and projects providing evidence for the synthesis

Table 1 Projects providing evidence for the synthesis

| **Project title** | **Funders and clients^1^** |
| --- | --- |
| Assessing the indirect effects of COVID-19 on essential health and nutrition services in selected rural and urban settings of Bangladesh | Funded by the Global Financing Facility, managed by the World Bank |
| Building Resilience in Ethiopia – Technical Assistance | FCDO and USAID |
| Centre for Disaster Protection | FCDO |
| District Health Systems Strengthening evaluation | Funded by BMGF, managed by UNICEF |
| Maintaining essential services after a natural disaster | FCDO |
| Policy and Institutions Facility – Nepal | FCDO |
| Real time assessment of UNICEF’s ongoing response to COVID-19 in eastern and southern Africa | UNICEF |
| The Bihar Technical Support Programme - learning grant | BMGF |

^1^ Funder indicated is for OPM input; some reports (such as COVID-19 intra-action reviews in Ethiopia) may also have been supported through other funding such as core government funds

Table 2 Report included in the synthesis

| **Report Title** | **Year** | **Country** | **Focus** | **Methods** | **Key organisational partners for report** | **Authors** |
| --- | --- | --- | --- | --- | --- | --- |
| **Building Resilience in Ethiopia – Technical Assistance** | | | | | | |
| Intra-Action Review On Public Health Preparedness And Response To Covid-19 In Ethiopia | 2020 | Ethiopia | Identifying challenges and best practices in the response to COVID-19 to inform adaptation of the current response - national | Document review, interviews with government staff, focus group discussions with government staff and experts, validation workshop with government | Ethiopia Ministry of Health, Ethiopian Public Health Institute, Regional Health Bureaus | Ministry of Health – Ethiopia, and Ethiopian Public Health Institute |
| Intra-Action Review On Public Health Preparedness And Response To Covid-19 In Gambella Region | 2021 | Ethiopia | Identifying challenges and best practices in the response to COVID-19 to inform adaptation of the current response – Gambella region | Document review, interviews with government staff, focus group discussions with government staff and experts, validation workshop with government | Ethiopia Ministry of Health, Ethiopian Public Health Institute, Regional Health Bureau | Gambella Region Health Bureau, COVID-19 Public Health Emergency Operation Centre, WHO and Partners |
| Intra-Action Review On Public Health Emergency Preparedness And Response To Covid-19 Sidama Regional Health Bureau | 2021 | Ethiopia | Identifying challenges and best practices in the response to COVID-19 to inform adaptation of the current response – Sidama region | Document review, interviews with government staff, focus group discussions with government staff and experts, validation workshop with government | Ethiopia Ministry of Health, Ethiopian Public Health Institute, Regional Health Bureau | Sidama Regional Health Bureau, Ministry of Health – Ethiopia and Ethiopian Public Health Institute |
| Health Preparedness And Response To Covid19 Report Oromia Regional State | 2021 | Ethiopia | Identifying challenges and best practices in the response to COVID-19 to inform adaptation of the current response – Oromia region | Document review, interviews with government staff, focus group discussions with government staff and experts, validation workshop with government | Ethiopia Ministry of Health, Ethiopian Public Health Institute, Regional Health Bureau | Oromia Regional Health Bureau, WHO, and BRE-OPM. |
| Intra-Action Review On Public Health Emergency Preparedness And Response To Covid-19 South Nations, Nationalities and People’s Regional Health Bureau | 2021 | Ethiopia | Identifying challenges and best practices in the response to COVID-19 to inform adaptation of the current response – SNNPR | Document review, interviews with government staff, focus group discussions with government staff and experts, validation workshop with government | Ethiopia Ministry of Health, Ethiopian Public Health Institute, Regional Health Bureau | SNNPR Health Bureau. |
| Intra-Action Review On Public Health Preparedness And Response To Covid-19 Somali Regional Health Bureau | 2021 | Ethiopia | Identifying challenges and best practices in the response to COVID-19 to inform adaptation of the current response – Somali Region | Document review, interviews with government staff, focus group discussions with government staff and experts, validation workshop with government | Ethiopia Ministry of Health, Ethiopian Public Health Institute, Regional Health Bureau | Somali Regional Health Bureau |
| Study to Assess the Effectiveness of the National Disaster Risk Management Commission’s Coordination role in recent disasters in Ethiopia | 2021 | Ethiopia | To assess the effectiveness of the National Disaster Risk Management Commission’s coordination role in recent disasters, including COVID-19, desert locusts, floods, drought, and conflict-induced displacement | Document review, interviews with national and subnational government, development agencies, NGOs and other experts | VNG Consulting | Bill Gray and Dr. Eleni Asmare |
| Rapid Regional Coordination System Review | 2020 | Ethiopia | To understand current emergency coordination mechanisms at the Regional and City level, and to identify challenges and areas for support (all experienced emergencies) | Interviews with subnational government | US Forest Service | OPM Building Resilience in Ethiopia project team and US Forest Service. |
| DRM Mainstreaming Gains in Key Ministries in Ethiopia | 2021 | Ethiopia | To assess progress in the level of DRM mainstreaming in selected lead sector institutions (all experienced emergencies) | Document review, interviews with senior government and development agencies, semi-structured to government | Multi-DREM Consulting Service PLC | Teshome Erkineh |
| Swan Evaluation: A Report On The Findings | 2021 | Ethiopia | Evaluation of an NGO consortium rapid response mechanisms for humanitarian support, including provision of essential humanitarian supplies in health, WASH, shelter and non-food items  (the SWAN project) | National and subnational interviews with government, development agencies, NGOs and community leaders, focus groups with beneficiaries | Monitoring Evaluation, Research and Quality improvement consultancy -  MERQ PLC (Ethiopia) | Jesse McConnell, Arpita Chakraborty, Erica Favretti, Donna Harris, Mayarani  Nurul Islami, and Fikralem Mezgebu |
| BRE Operational Research: Assessing the efficiency and effectiveness of emergency health financing mechanisms in Ethiopia | 2021 | Ethiopia | To examine the current emergency health and nutrition financing system, with case studies on malaria, cholera, COVID-19 and nutrition shocks, including drought and floods | Document review and analysis of secondary data | Partnership and Cooperation Directorate, Federal Ministry of Health | Donna Harris, Ageazit Teka, Zoma Mesfin, Edom Betru, Ermias Dessie and Tesfaye Mesele |
| **Maintaining essential services after a natural disaster** | | | | | | |
| Initial Covid19 Responses In Bangladesh, Kenya, Pakistan, Sierra Leone, And Uganda | 2020 | Bangladesh, Kenya, Pakistan, Sierra Leone, And Uganda | Rapid situation analyses on the initial response to COVID-19 in the first few months of the outbreak, to identify learning around the national ability to respond to shocks | Document review and key informant interviews with government, development agencies and other experts |  | Debbie Hillier, Tom Newton-Lewis, Rithika Nair, and Christoph Larsen |
| COVID-19 Response: Rapid country study: Pakistan. | 2020 | Pakistan | Initial documentation of the early government response to COVID-19, to identify lessons and opportunities for providing support | Document review and key informant interviews with government, development agencies and other experts |  | Syed Zulfiqar Ali |
| COVID-19 Response: Rapid country study: Kenya. | 2020 | Kenya | Initial documentation of the early government response to COVID-19, to identify lessons and opportunities for providing support | Document review and key informant interviews with government, development agencies and other experts |  | Akaco Ekirapa |
| Sierra Leone’s response to COVID-19 | 2020 | Sierra Leone | To describe initial government efforts to respond to COVID-19, and to understand how COVID-19 is affecting health and non-health aspects of life. | Document review, interviews with government and NGOs, virtual attendance at EOC inter-pillar meetings, survey of community members |  | Kevin Grieco, Yasmina Yusuf, and Niccoló Meriggi |
| The effectiveness of the Sierra Leone health sector response to health shocks: Evidence from the COVID-19 perception survey. | 2020 | Sierra Leone | To examine perceptions of national and district government staff, development agencies, health workers and members of COVID-19 coordinating committees on the COVID-19 response, with specific focus on service delivery, leadership and governance, health workforce, and community ownership and participation | Survey of 303 respondents using computer assisted interviewing | Institute for Development, International Growth Centre, Dalan Development Consultants and College of Medicine and Allied Health Sciences | Philip S. Amara, Fredline A M’Cormack-Hale, Mohamed Kanu, Regina Bash-Taqi, and Alhassan Kanu |
| Beyond the state: The role of traditional leaders in COVID-19 | 2020 | Sierra Leone | Rapid scoping study to describe district-level coordination and implementation of policy relating to COVID-19, including the role of traditional leaders in the response | Interviews with district government, traditional leaders, and health professionals in Kono district | International Growth Centre, Dalan Development Consultants and College of Medicine and Allied Health Sciences | Kevin Grieco |
| Climate shock responsiveness of the Kenya health system | 2020 | Kenya | To examine how county-level actors prepare for and respond to climate shocks, focusing on drought and flood events in 2019 in the northern Kenyan counties | Document review, secondary data analysis, and interviews with subnational government, development agencies and health workers | Centre for Humanitarian Change | Matt Fortnam, Peter Hailey, Emily Mbelenga, Nancy Balfour, Stephen  Odhiambo, and Elijah Odundo |
| Innovation history of the CMAM Surge approach Towards a shock-responsive health system in Kenya | 2020 | Kenya | To understand the processes, enablers and barriers to the emergence and development of Community-based Management of Acute Malnutrition (CMAM) surge, and its perceived potential and limits to building health system shock-responsiveness | Document review, interviews with national and subnational government, development agencies and NGOs, health workers | Centre for Humanitarian Change | Matt Fortnam, Peter Hailey, Nancy Balfour, Katy Sheen and Rebecca Lea |
| Response and Preparedness for Essential Health and Nutrition Services During Disasters in Pakistan | 2021 | Pakistan | To examine health system preparedness and response to shocks (floods, droughts and COVID-19), including national and subnational government mechanisms, experience with recent shocks, and community needs and resilience | Document review and analysis of secondary data, key informant interviews with national and provincial government, NGOs, health workers, development agencies, community stakeholders and other stakeholders, focus groups with CHW and community members | Department of Community Health Sciences Aga Khan University, Karachi (Pakistan) | Zafar Fatmi, Rabia Najmi, Nousheen Akber Pradhan, Zarak Ahmed, Minhaj Qidwai, Sana Hyat, and Sania Khursheed |
| **Assessing the Indirect Effects of COVID-19 on Essential Health and Nutrition Services in selected rural and urban settings of Bangladesh.** | | | | | | |
| Assessing the Indirect Effects of COVID-19 on Essential Health and Nutrition Services in selected rural and urban settings of Bangladesh | 2021 | Bangladesh | To understand the impacts of COVID-19 on delivery of essential health services by government and private providers in urban and rural areas, including demand and supply side challenges and coping strategies | Secondary data analysis, interviews with health service providers, community leaders, and key informant stakeholders (including government, NGOs and academics), focus groups with service providers | Development Research Initiatives (dRi) | Muhammod Abdus Sabur, Nazme Sabina, and Adiba Khaled |
| **Policy and Institutions Facility – Nepal** | | | | | | |
| Learning from Nepal NRA to inform the National Disaster Risk Reduction and Management Authority | 2019 | Nepal, and review of experience in Pakistan, India, and Bangladesh | To identify learning from the National Reconstruction Authority (NRA) experience, to inform development of the new National Disaster Risk Reduction and Management Authority (NDRRMA).  . | Literature review, consultations with NRA officials, other stakeholders familiar with the NRA, development agencies and NGOs, review of experience with reconstruction and disaster management in other South Asian countries (India, Pakistan, Bangladesh). |  | Dinanath Bhandari and Christopher Hodder |
| Disaster risk reduction and management in Nepal: Delineation of roles and Responsibilities | 2020 | Nepal, and review of experience in Pakistan, India, Bangladesh | To provide policy recommendations on the delineation of roles, responsibility and accountability of federal, provincial and local governments for disaster risk reduction and management, including understanding existing subnational capacities | Document review, consultations with local government leaders and bureaucrats, development agencies and other experts |  | Dinanath Bhandari, Sanchita Neupane, Peter Hayes, Bimal Regmi and Phil Marker |
| **The Bihar Technical Support Programme - learning grant** | | | | | | |
| Managing the COVID-19 Crisis: Insights from Kerala | 2021 | India | To examine Kerala government’s early response to COVID-19, including mechanisms used to control the pandemic | Document review, a small number of interviews with representatives from senior government, self-help groups and health workers |  | Divya Nambiar, Arpana Kullu and Radhika Arora |
| **Real time assessment (RTA) of UNICEF’s ongoing response to COVID-19 in eastern and southern Africa:** | | | | | | |
| Real time assessment (RTA) of UNICEF’s ongoing response to COVID-19 in eastern and southern Africa: COVID-19 vaccine supply and rollout | 2021 | Ethiopia, Rwanda, South Africa, South Sudan | To assess UNICEF’s support to COVID-19 vaccine supply in the eastern and southern Africa region, in order to support learning for ongoing and future support | Document review, interviews with national and local government and development partners |  | Kate Gooding, Jayne Webster, Nicola Wiafe and Vimal Kumar |
| **Evaluation of UNICEF’s District Health Systems Strengthening Initiative** | | | | | | |
| Evaluation of UNICEF’s District Health Systems Strengthening Initiative | 2021 | Uganda (evaluation also covered Kenya, Malawi, Tanzania) | To evaluate a cross-country programme to strengthen subnational health planning and management | Document review, interviews with national and local government, implementing partners and other stakeholders, focus group with local government |  | Kate Gooding, Gabrielle Appleford, and Nicola Wiafe. |
| **Centre for Disaster Protection** | | | | | | |
| Opportunity Cost of COVID-19 emergency expenditure reallocations: inception report | 2020 | Pakistan, South Africa | To examine the costs and benefits of using ex-post public budget reallocations, as a financing instrument for disaster response | Document review and analysis of secondary data |  | Stephanie Allan and Dayna Connolly |

1 Organisations are indicated for reports where other organisations beyond OPM were involved in co-producing the research and analysis. The wider projects also involve other partners; those listed here are restricted to organisations involved in producing the reports used in the synthesis.
