# Supplementary material for: How can we strengthen partnership and coordination for health system emergency preparedness and response? Findings from a synthesis of experience across countries facing shocks
Source: BMC Health Serv Res. 2022 Nov 29;22:1441. doi: 10.1186/s12913-022-08859-6 (PMC9706990; doi:10.1186/s12913-022-08859-6)
Supplement: Supplementary file 2 — Additional file 2. Coding framework. [file 12913_2022_8859_MOESM2_ESM.docx]

**Additional file 2: coding framework**

The framework below was used for initial coding, with further themes added inductively, and grouping of themes reviewed and adjusted over time and during later analysis.

**Private sector partnership (including the private health sector and other private organisations)**

- Forms of collaboration/partnership between private sector and government (e.g. what partnership was undertaken, when, what for)
- Roles played by private sector
- Benefits/strengths of private sector collaboration and how this affects resilience
- Gaps or disadvantages/challenges around private sector collaboration and how this affects resilience

**Development agency partnership**

- Forms of collaboration/partnership with development agencies
- Roles played by development agencies
- Benefits /strengths of development agency partnership and how this affects resilience
- Gaps or disadvantages/challenges around development agency partnership and how this affects resilience

**Local leaders’ partnership:**

- Forms of collaboration/partnership with local leaders
- Roles played by local leaders
- Benefits /strengths of development agency partnership and how this affects resilience
- Gaps or disadvantages/challenges around development agency partnership and how this affects resilience

**National Coordination structures**

- What structures exist

*For all below: information on whether these issues are present, positives/negatives and how this affects preparedness and response*

- Government leadership
- Accountability
- Capacity of coordination structures (e.g. capacity of secretariat/participants to coordinate, considering time, skills, budget, infrastructure, etc.)
- Mandate and authority of coordinating structures and lead organisations
- Clarity on roles – of the coordination body and individual partners
- Balance of health sector and other actors
- Use of pre-existing or new coordination structures
- Functioning of structures and coordination pre-disaster
- Information sharing
- Other strengths
- Other weaknesses

**Subnational structures**

- What structures exist
- *For all below: information on whether these issues are present, positives/negatives and how this affects preparedness and response*
- Government leadership
- Accountability
- Capacity of coordination structures
- Coordination and partnership between national/subnational levels
- Clarity on roles – of the coordination body and individual partners
- Balance of health sector and other actors
- Use of pre-existing or new coordination structures
- Functioning of structures and coordination pre-disaster
- Information sharing
- Other strengths
- Other weaknesses

**Consideration of gender, equity and social inclusion in coordination**

- Strengths
- weaknesses

Research gaps – gaps in evidence identified in the reports
